# Deciphering trait diversity in rainy-season onion: a comprehensive evaluation of morphological, nutritional, biochemical, and molecular attributes

**DOI:** 10.3389/fpls.2026.1900180

**Published:** 2026-08-12

**Authors:** Manisha Shivaji Jadhav, Bharat Tukaram Patil, Kalpana Narayan Dahatonde, Sharmila Rajaram Shinde, Amol Vilas Kshirsagar, Kalyani Gorrepati, A. Thangasamy, Sanket J. More, Hem Raj Bhandari, Bhushan Bibwe, Rahul Yadav, Yogesh P Khade

**Affiliations:** 1 Mahatma Phule Krishi Vidyapeeth, Rahuri, Maharashtra, India; 2 Indian Council of Agricultural Research (ICAR)-Directorate of Onion and Garlic Research, Rajgurunagar, Pune, Maharashtra, India; 3 ICAR-Central Institute of Post-Harvest Engineering and Technology, Ludhiana, Punjab, India

**Keywords:** bio-fortification, genetic diversity, mineral composition, nutritional profiling, trait-specific breeding

## Abstract

The present study employed a comprehensive multi-traits-based approach to evaluate thirty-one onion genotypes for morphological, biochemical, mineral, and molecular traits with the objective of identifying nutritionally superior genotypes suitable for rainy-season cultivation. Substantial genotypic variability was observed for major quality traits, including total phenols (172.76–446.73 mg/100 g FW), flavonoids (138.40–627.07 mg/kg FW), total antioxidants (FRAP) (1284.15–5538.31 µmol/100 g FW), pyruvic acid (1.33–11.65 µmol/g FW), total sugars (2.42–6.76%), reducing sugars (1.28–3.91%), non-reducing sugars (0.42–4.86%), total soluble solids (8.3–14.3°Brix), anthocyanin (2.87–44.18 mg/100 g DW), moisture content (85.36–89.13%), dry matter (10.87–14.64%), and mineral composition. Multi-trait donor genotypes combining yield, biochemical and mineral traits were identified through integrated PCA, PCA biplot and clustering analyses, with DOGR-2103 emerging as a key superior genotype. DOGR-2104 and DOGR-2101 also exhibited desirable combinations for yield and quality traits, while DOGR-2119 and DOGR-2114 were associated with quality and mineral traits. SSR marker analysis further identified RHRO-2022–10 and DOGR-2107 as genetically distinct genotypes with potential utility in molecular breeding and hybridization programs. In addition, principal component analysis revealed strong positive associations among sugars, total soluble solids, dry matter, and antioxidant-related traits, indicating their utility in selecting genotypes suitable for enhanced nutritional quality, processing efficiency and storability. In contrast, moisture content exhibited an inverse relationship with most of the quality attributes, highlighting the importance of developing low-moisture and high dry-matter rainy-season genotypes for improved storability and processing quality. Further, the identified trait-specific superior and genetically diverse donors represent valuable breeding foundation for developing nutritionally rich high quality onion cultivars suited to rainy-season. It also highlights the potential of bio-fortification for enhancing market value and consumer preference for both fresh consumption and processing in onion.

## Introduction

Onion (*Allium cepa* L., 2n = 2x = 16), belonging to the family Amaryllidaceae, is one of the most widely cultivated vegetable crops worldwide due to its culinary and nutritional importance. In India, onion is grown across diverse agro-climatic regions, with Maharashtra contributing nearly 43% of the total national production, followed by Madhya Pradesh (16%), while Karnataka and Gujarat each account for approximately 9%. Onion cultivation in the country is distributed across three distinct cropping seasons, namely rainy-season (June – October), post-rainy season (August – January) and winter season (October – April), ensuring year-round availability. Besides its culinary importance, onion is widely valued for its nutritional attributes, unique aroma, flavor, pungency, and preservative and processing suitability, which collectively contribute significantly to its dietary, industrial and commercial importance (Bhattacharjee et al., 2013; Bhandari et al., 2026).

Onion is a rich source of phenolic acids, including gallic, ferulic, p-coumaric and caffeic acids, which contribute significantly to its bioactive composition and play an important role in its nutraceutical and health promoting properties (Teshika et al., 2019; Bibi et al., 2022; Gupta et al., 2025b). Pungency in onion is primarily associated with pyruvate formation, which results from alliinase-mediated hydrolysis of sulfur-containing compounds and is commonly quantified using the DNPH method. Although environmental conditions and post-harvest storage influence flavor intensity, genetic factors play a predominant role in regulating sulfur metabolism and determining pungency levels (Randle, 1992; Randle et al., 1995). Sulfur availability further contributes to variation in pungency expression (de Oliveira et al., 2025). The characteristic red coloration in onion bulbs is attributed to anthocyanins, predominantly cyanidin and peonidin glucosides, occurring in both acylated and non-acylated forms (Mazza and Miniati, 1993). Red onions are reported to contain a diverse range of anthocyanins, constituting a significant proportion of total flavonoids, with high concentrations on a fresh weight basis (Slimestad et al., 2007). These pigments also contribute to functional properties of onion (Geetha et al., 2011). In contrast, white onions exhibit comparatively lower flavonoid accumulation, reflecting limited pigmentation and reduced antioxidant potential (Herrmann, 1976). Therefore, red onion genotypes possessing higher anthocyanin and flavonoid content may serve as valuable genetic resources for developing nutritionally enriched cultivars with enhanced antioxidant and functional food properties.

In addition to these bioactive compounds, onion also serves as an important dietary source of essential minerals associated with human health and nutritional security. Onion is a notable source of essential minerals such as calcium, potassium, and iron, which are critical for bone development, muscle function, and hematological health (Mahmood et al., 2021). The favorable potassium-to-sodium ratio of onion makes it particularly beneficial for individuals with hypertension (Edet et al., 2015; Singh et al., 2023). Preclinical and clinical studies have consistently highlighted the antioxidant, anti-inflammatory, antimicrobial and anticancer properties of onion bioactives, supporting their potential as therapeutic and preventive agents. Bioactive compounds such as flavonoids (including quercetin), phenolics and sulfur-containing compounds not only contribute to the medicinal properties of onion but also enhance its nutritional value. Furthermore, the bioavailability and molecular profiling of these compounds suggest promising applications in the development of functional foods and pharmaceutical interventions (Gupta et al., 2025b). These nutritional and functional attributes have gained increasing importance in modern breeding programs aimed at developing health-promoting vegetable cultivars.

The molecular markers provide a highly reliable approach for genetic diversity assessment, as they remain largely unaffected by environmental noise compared with phenotypic and biochemical traits while enabling rapid and scalable DNA-level analysis (Melchinger et al., 1991). Among the available molecular marker systems, simple sequence repeat (SSR) markers are widely preferred due to their high polymorphism, multi-allelic nature, co-dominant inheritance, reproducibility, and compatibility with automated high-throughput genotyping platforms (Kalia et al., 2011). The information on bulb quality traits such as total soluble solids (TSS), total phenolic content, antioxidant capacity, and nutrient composition, which are important indicators of functional food potential, is still in its paucity (Islam et al., 2017). Existing studies have reported fragmented information on the nutritional composition of onion (Kale and Ajjappalavara, 2015; Nemtinov et al., 2021; Chandel et al., 2024); however, comprehensive genotype-specific profiling of major macro- and micro-nutrients in conjunction with biochemical and molecular traits remains largely unexplored.

In recent years, growing consumer awareness regarding functional foods and nutritional security has increased the demand for onion cultivars with superior biochemical and nutritional quality traits (Singh and Khar, 2024). However, despite the economic importance of rainy-season onion cultivation, breeding programs have largely focused on yield and disease resistance, with comparatively limited emphasis on nutritional and functional quality enhancement (Mahajan et al., 2021).

The distinct agro-climatic conditions prevailing during the rainy-season may substantially influence nutrient uptake, translocation and assimilate partitioning in onion plants compared with other seasons, thereby highlighting the importance of season-specific evaluations of nutritionally important traits. Moreover, the productivity of rainy-season onions is substantially less as compared to post-rainy and winter season cultivars posing additional challenges for simultaneous improvement of yield and bulb quality. Therefore, a comprehensive and integrated assessment of genetic variation in macro- and micronutrient composition together with associated biochemical and molecular attributes is essential for accelerating the development of nutritionally enriched onion cultivars adapted to rainy-season cultivation.

In this context, the present study undertook a multidimensional trait-based profiling of rainy-season onion genotypes through systematic evaluation of genotype-specific macro and micro-nutrient profiling in conjunction with key biochemical and molecular traits. The findings will provide valuable baseline information for trait-oriented breeding programs and facilitate the development of nutritionally superior onion cultivars, thereby contributing to improved dietary quality and sustainable onion production.

## Materials and methods

### Genotypes and reagents

The plant material consisted of thirty-one diverse onion genotypes (**Supplementary Table 1**) specific for rainy-season cultivation, evaluated during the year Kharif-2024, including two commercially adopted varieties showing morphological diversity for bulb color, shape, size etc. These collections were obtained from Field Gene Bank of ICAR-Directorate of Onion and Garlic Research (DOGR), Pune, Maharashtra and Mahatma Phule Krishi Vidyapeeth, Rahuri, Maharashtra, India. This farm is situated at 18.84° N latitude and 73.88° E longitude. The geographical location falls under subtropical dry humid climate. All the agronomical practices recommended by ICAR-DOGR for rainy-season onion production was followed. All biochemical analyses were carried out using analytical grade chemicals procured from Himedia. High purity standards (*>*98% purity) were procured from Sigma-Aldrich. The experiment was conducted using a randomized block design with three replications, each replication consisting of about 400 plants per plot for each genotype.

### Morphological and nutritional assessment of bulb

After harvesting, 5 bulbs per plot were randomly used for average bulb weight (g), equatorial diameter (cm), polar diameter (cm), neck thickness (mm) and TSS (°Brix). For the nutritional analysis, ten bulbs were collected from each replication and fine homogeneous paste was prepared by using mixer grinder (Philips, Model-HL7699/00) for biochemical analysis. The samples were ground and stored at -20°C for subsequent nutritional composition analysis.

### Total soluble solids (TSS)

From each genotype, 5 bulbs were randomly selected. Juice was extracted using a blender, and filtered through a strainer to obtain a clear extract. A drop of the filtrate was placed on the prism of a hand held Refractometer (Avantor, India), ensuring even coverage, the °Brix value was recorded. The prism was also cleaned with distilled water and dried with a soft, lint-free cloth after each reading to prevent cross-contamination between samples.

### Moisture content and dry matter

Approximately 100 g of chopped onion sample was taken in petri plates. They were dried in hot air oven at 37 - 65°C up to a constant weight is observed. Moisture content was calculated by subtracting dry weight from initial sample weight on percentage basis (Gupta et al., 2025a).

### Total sugar, reducing and non-reducing sugar

The total sugar was estimated by the method described by Yemm and Willis (1954) using the Anthrone reagent with some minor modifications and the absorbance measured at 630 nm. The reducing sugar content was determined according to the Nelson (1944) and Somogyi (1952) method with certain modifications suggested by Gupta et al. (2024). The absorbance was measured at 540 nm using a spectrophotometer (Spectro Star Nano, BMG Labtech, (Germany). The non-reducing sugar values were calculated by using following formula:


Non−reducing sugars(g 100 g−1 FW)=Total sugars−Reducing sugars

### Biochemical analysis

#### Anthocyanin

Total monomeric anthocyanin content was determined using the pH differential method, which is based on the pH-dependent structural transformation of anthocyanins from the colored oxonium ion form at pH 1.0 to the colorless hemiketal form at pH 4.5, following the protocol described by Lee et al. (2005) with modifications as reported by Krithika et al. (2020).

#### Total phenols

The total phenolic content was determined using the Folin–Ciocalteu assay according to the method of Singleton and Rossi (1965) with some modifications suggested by Ateeq et al. (2023). A calibration curve was prepared using gallic acid as the standard (R² = 0.9996), and the total phenolic content was reported as gallic acid equivalents per 100 grams of fresh weight.

#### Total antioxidants (FRAP)

Total antioxidant activity was measured using the FRAP assay, which detects the formation of a blue to purple color as the ferric tripyridyltriazine (FeIII-TPTZ) complex is reduced, following the procedure of Benzie and Strain (1999) with some modifications suggested by Sagar et al. (2020). The results were calculated using a synthetic ascorbic acid analog as the standard and expressed as micromoles per gram of fresh weight.

#### Pyruvic acid

Pyruvic acid quantification was done by following 2,4-dinitrophenyl hydrazine (DNPH) reagent method described by Anthon and Barrett (2003), incorporating a minor modification to the original Schwimmer and Weston (1961) protocol.

#### Flavonoids

The extract prepared for phenol was used for flavonoid. Total flavonoid content was estimated by spectrophotometer mentioned by Lombard et al. (2002).

### Mineral analysis

#### Nitrogen (N), phosphorous (P), sulfur (S) and potassium (K)

Total nitrogen was estimated by the Kjeldahl digestion method (Parkinson and Allen, 1975).

Phosphorus was determined colorimetrically using the Vanadate-Molybdate reagent (Jackson, 1973). Sulfur was measured with barium chloride reagent (Tabatabai and Bremner, 1970) on a BMG Labtech Spectrostar Nano spectrophotometer (Germany) at 420 nm (P) and 440 nm (S), respectively. Potassium was quantified by flame photometry (Chapman and Pratt, 1961) using a Micro Controller Flame Photometer (Labtronics, Model LT-6715).

#### Copper (Cu) and manganese (Mn)

The copper and manganese were analyzed using an Atomic Absorption Spectrophotometer (AAS) (Perkin Elmer Singapur, Model-Pin AAcle 500) as described by Zasoski and Burau (1977).

#### Calcium and magnesium

Calcium and magnesium were determined by titration method as described by Jackson (1967).

#### Boron (B)

Boron in plant samples was measured through dry ashing. The concentration of boron was measured by dry ashing (Chapman and Pratt, 1961) and subsequent measurement of boron by colorimetric method with using Azomethine-H (Bingham, 1982).

### Molecular diversity using SSR marker

#### DNA extraction

Genomic DNA was extracted from 100 mg leaf sample for each of thirty-one onion genotypes using CTAB (Doyle and Doyle, 1987; modified by Murray and Thompson, 1980). Quality was assessed by NanoDrop (A260:A280) and 0.8% agarose gel. DNA was diluted to 25 ng/µL and stored at -20°C.

#### SSR marker amplification

A set of SSR primer pairs was selected and synthesized by Eurofins Genomics India. PCR amplification was performed in a 20 µL reaction volume containing 10X reaction buffer, 50 ng template DNA, 1.5 mM MgCl_2_, 0.2 mM of each dNTP, 0.2 µM of each primer, and 5 U of Taq DNA polymerase. The amplification was carried out in a Bio-Rad Laboratories iCycler thermal cycler with an initial denaturation at 94 °C for 4 minutes, followed by 38 cycles of denaturation at 94 °C for 45 seconds, annealing at primer specific annealing temperature (**Table 1**) for 1 minute and final extension at 72 °C for 10 minutes. The amplified PCR products were resolved on a 3% agarose gel through electrophoresis. Band sizes were estimated using a 1 kb Plus DNA ladder from Thermo Fisher Scientific, and the gels were documented using a gel documentation system.

**Table 1:** Polymorphic information content of various markers.

Sr. no.	Primer name	Primer F/R	Primer sequence 5’-3’	Tm (°C)	Allele size range	PIC
1	ACM 53	ACM 53 -F	CTGGGCTCTTTTGTTCATCC	55.2	190-450	0.24
		ACM 53-R	ATGGTGGAGGTATGTGAGGG			
2	ACM 54	ACM 54-F	GAGTGAGAGGGGAAATGGAA	50.3	210-470	0.41
		ACM 54-R	AAAGATGGTTTGTTGGTGGC			
3	ACM 61	ACM 61-F	GCGTTTGCTGAGAGATTACGA	50.3	200-700	0.32
		ACM 61-R	TTTCTTGCTGATGATGCTGC			
4	ACM 78	ACM 78 -F	CGCAGAATCTCGTCCTTTTT	50.3	290-600	0.44
		ACM 78-R	AATGGTTTGGAGGTCAGTCG			
5	ACM 107	ACM 107-F	CCTTCATTCCCAAAGCACAT	55.0	210-390	0.17
		ACM 107-R	GCGATAAAGAGGGACAGCAG			
6	ACM 101	ACM 101-F	CCTTTGTAACCAAATCCGA	55.0	200-300	0.28
		ACM 101-R	CTTGTTGAGAAGGAGGACGC			
7	ACM 9	ACM 9-F	GCAACGGTAGAAGAACCTGC	55.0	250-700	0.57
		ACM 9-R	AACCTCTTTTGGTGCCTCCT			
8	ACM 16	ACM 16-F	ATGGAAGCCTCGGGTCTG	55.0	210-700	0.43
		ACM 16-R	GCCGTAAGTCGAGGGTAGAA			
9	ACM 18	ACM 18-F	GGGGAATGGTGGAGAATAGA	55.0	200-700	0.49
		ACM 18-R	AACAGAGGCAAGAGGAGCG			
10	ACM 77	ACM 77-F	AAATTATGGGCCACCTCCTC	52.3	190-650	0.50
		ACM 77-R	CAAGATTGTCGACTCCCCAT			
11	ACM 63	ACM 63 -F	TCACGCACATACCTCTCTCG	55.0	190-230	0.50
		ACM 63-R	TGCCAAGGTGCATAAAAACA			
12	ACM 38	ACM 38-F	ATGCCAGACTACGACAACGA	55.0	200	0.12
		ACM 38-R	ACGCCTACCAACCTTCAATG			
13	ACM 93	ACM 93-F	GCCAACAGTTTTCGTAAGTTGA	50.3	150	0.45
		ACM 93-R	ATTCTCTTCGGCTTTCGTGA			
14	ACM 4	ACM 4-F	TCGTTCTTTAGAACACTGTAGGAA	58.0	200	0.40
		ACM 4-R	TGTCGGCGGATATAGTGACA			
15	ACM 45	ACM 45-F	AAAACGAAGCAACAAACAAAA	45.1	270	0.54
		ACM 45 -R	CGACGAAGGTCATAAGTAGGC			
16	ACM 5	ACM 5-F	CGCTTCAGCAGTGAGTTGTT	55.0	200	0.62
		ACM 5-R	CGCTTCAGCAGTGAGTTGTT			
17	ACM 6	ACM 6 -F	GCAGTTCTCCCTTTGTAAAATCA	55.0	200	0.58
		ACM 6-R	GTGATGGATGAGTGGATGGA			
18	ACM 91	ACM 91-F	TCTCCTCCTCTAACCAGCCA	55.0	190	0.50
		ACM 91-R	GGTGCTCCAGTTGAGCTTTC			
19	ACM 23	ACM 23-F	CCCACTCGCTTTCATACTGC	55.0	400	0.24
		ACM 23-R	CCACTCCTCTTACTGCTTCCC			
20	ACM 68	ACM 68-F	CGAAGGTGAAGGTGTACGGT	55.0	200	0.80
		ACM 68-R	CAAATGGCTGCAATAAGCAA			
21	ACM 65	ACM 35-F	GCTCTGATGGACGATGGTTC	55.0	200	0.35
		ACM 65-R	TTCTTGCCATCTTTGTCGGT			
22	ACM 115	ACM 115-F	TCCATCTATGCCTCTGCCAC	52.3	220	0.30
		ACM 115-R	CTATTCTTCCACTGGGGCAA			
23	ACM 52	ACM 52-F	CAGCAGCAACAAAGAATG	55.0	230	0.12
		ACM 52-R	CTGGGGAGAATGAGAAGC			
24	ACM 81	ACM 81-F	CTGAAAAGAAACCCGCAGAG	55.0	210	0.12
		ACM 81-R	TCAGGATGCACTTGCTTCAG			
25	ACM 80	ACM 80-F	GCATTATGCAGTAACGGGCT	55.0	200	0.06
		ACM 80-R	GCAGCAGCATTTGATTGAAC			
26	ACM 56	ACM 56-F	TGTGTGGAAATCAGTAGGCT	52.3	250	0.06
		ACM 56-R	ATGACAACAGAACCGCTGG			
27	ACM 34	ACM 34- F	CACCTTGGACCGTGAAGAAC	55.0	250	0.06
		ACM 34-R	CTGCTGTTTGGAGATGTGGA			
28	ACM 104	ACM 104-F	ACGATTGTTTAATCGTCCGC	55.0	200	0.06
		ACM 104-R	TATTCCAGCAGCTGTCG			
29	ACM 33	ACM 33-F	ATCATCGTCCTCGTCCTCAT	55.0	250	0.06
		ACM 33-R	CCTTCTCCCCATTCTCTTCC			

#### SSR analysis

Bands were scored as presence (1) or absence (0). Polymorphic information content (PIC) was calculated and Jaccard’s similarity coefficients were determined using NTSYS-pc v2.02i (Rohlf, 1998). A UPGMA dendrogram was constructed (Bhandawat et al., 2015).

#### Statistical analysis

All statistical analyses were conducted using R software. The estimation of key genetic parameters- including genotypic variance (Vg), phenotypic variance (Vp), environmental variance (Ve), coefficient of variation (CV), genotypic coefficient of variation (GCV), phenotypic coefficient of variation (PCV), broad-sense heritability (H²), and genetic advance (%)- was performed using the R-based web application GRAPES 1.1.0 (https://www.kaugrapes.com/home), following the methodology of Gopinath et al. (2020). For the analysis of biochemical and mineral data, R software version 4.5.5 was used with statistical significance assessed at the 95% confidence level. The correlation coefficients and correlograms for selected bulb traits were calculated using Pearson’s method within the GRAPES platform at a significance level of 0.05. Additionally, heat map-based clustering and principal component analysis (PCA) and PCA biplot for the thirty-one genotypes, based on morphological, biochemical and mineral parameters by using R software.

## Results

### Assessment of genetic variability

Genetic variability parameters, including genotypic coefficient of variation (GCV), phenotypic coefficient of variation (PCV), broad-sense heritability (H²), and genetic advance as percent of mean (GAM), were estimated to assess the extent of genetic variability and effectiveness of selection among the evaluated onion genotypes. A comparatively higher difference between PCV and GCV values indicated greater environmental influence on trait expression. In the present study, high GCV and PCV values (>20%) were recorded for equatorial diameter, neck thickness, average bulb weight, yield per plot, and yield per hectare indicating the presence of substantial genetic variability for these economically important traits (**Table 2**; **Supplementary Figure 1**). Moderate GCV and PCV values (10-20%) were observed for polar diameter and number of leaves per plant, whereas low variability was recorded for plant height and total soluble solids (TSS**).** The relatively narrow differences between GCV and PCV observed for most traits suggested limited environmental influence and greater contribution of genetic factor towards phenotypic expression. Variation in bulb morphology among the studied genotypes was presented in **Supplementary Figures 2A–C**. In the present investigation, high heritability (*>*60%) estimates were observed for most of the traits studied except the number of leaves per plant. High heritability coupled with high GAM (*>*20%) was recorded for most yield-related and bulb-related traits, indicating the predominance of additive gene action and greater scope for effective phenotypic selection. In contrary, plant height exhibited high heritability associated with low GAM, suggesting comparatively lower expected genetic gain despite high heritable variation. However, number of leaves per plant exhibited low heritability coupled with high GAM, indicating possible environmental influence and non-additive gene effects. Similarly, TSS exhibited high heritability with low GAM, suggesting limited scope for direct selection despite strong genetic control.

**Table 2:** Genetic, phenotypic and environmental variance, GCV (%), PCV (%), heritability and GAM for important bulb and plant growth traits.

Traits	Genetic variance	Phenotypic variance	Environmental variance	Coefficient of variation (%)	GCV (%)	PCV (%)	Heritability	GAM (%) (i=5%)
Plant height	22.63	33.86	11.23	5.46	7.74	9.47	66.8	13.04
Number of leaves per plant	1.08	1.94	0.86	11.95	13.39	17.95	55.7	20.59
Equatorial diameter	1.05	1.21	0.16	9.34	23.97	25.73	86.8	46.01
Polar diameter	0.26	0.33	0.06	6.43	13.25	14.73	81.00	24.57
Neck thickness	4.27	5.77	1.50	15.46	26.07	30.31	74.00	46.20
Average bulb weight	379.10	389.63	10.54	6.29	37.72	38.24	97.3	76.64
Yield per plot	50.67	53.81	3.14	11.37	45.66	47.06	94.2	91.28
Yield per hectare	83.38	88.02	4.64	9.45	40.04	41.14	94.7	80.28
TSS	1.06	1.33	0.27	9.85	8.79	9.85	79.7	16.17

### Correlation for bulb quality parameters

**Figure 1:**
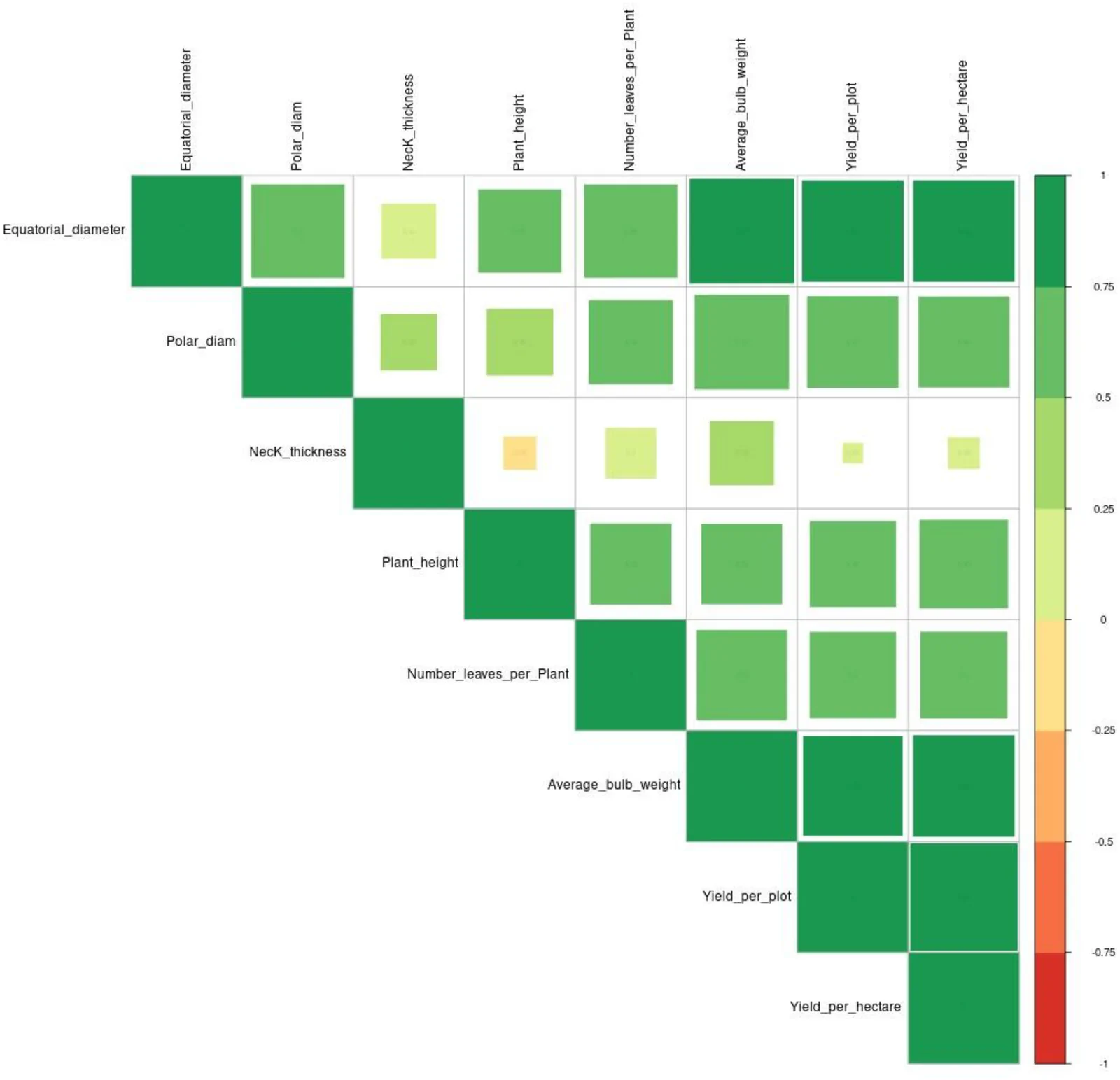
Pearson correlation showing relationship among economically important bulb quality and yield traits. (The color indicates values of correlation coefficient and size of the square is proportional to the magnitude of the correlation coefficient).

Correlation analysis among key traits was performed to understand the inter-relationship among economically important characteristics and also to identify traits useful for indirect selection in onion breeding program. The analysis revealed highly significant positive associations among major growth and yield attributes (**Figure 1**; **Table 3**). Equatorial diameter exhibited significant positive correlation with polar diameter, plant height, number of leaves per plant, average bulb weight, yield per plot and yield per hectare. Similarly, polar diameter was showed significant and positive correlation with plant height, number of leaves per plant, average bulb weight, yield per plot and yield per hectare. The plant height was also positively correlated with number of leaves per plant, average bulb weight, yield per plot and yield per hectare, while number of leaves per plant exhibited significant positive correlations with bulb weight and yield parameters. Among the yield-contributing traits, average bulb weight showed strong positive correlation with yield per plot and yield per hectare, whereas the strongest positive correlation was observed between yield per plot and yield per hectare. The observed positive interrelationships among bulb size, vegetative growth, and yield traits suggest that improvement in one trait may simultaneously enhance overall bulb productivity, thereby facilitating indirect selection for yield improvement in onion breeding programs. On the other hand, neck thickness exhibited weak or non-significant correlations with most of the studied traits. Neck thickness showed weak positive correlation with equatorial diameter and polar diameter, whereas weak and non-significant association was observed with number of leaves per plant, yield per plot and yield per hectare. A weak negative and non-significant correlation between neck thickness and plant height further indicated the limited contribution of neck thickness toward yield enhancement under the present experimental conditions.

**Table 3:** Correlation between economically important onion morphological traits estimated using Pearson’s correlation coefficient.

Traits	Plant height	Number of leaves per plant	Equatorial diameter	Polar diameter	Neck thickness	Average bulb weight	Yield per plot	Yield per hectare
Plant height	1.000	0.525***	0.549***	0.352***	-0.085	0.517***	0.591***	0.625***
Number of leaves per plant	0.525***	1.000	0.694***	0.564***	0.203	0.652***	0.595***	0.600***
Equatorial diameter	0.549***	0.694***	1.000	0.698***	0.238*	0.874***	0.824***	0.822***
Polar diameter	0.352***	0.564***	0.698***	1.000	0.253*	0.714***	0.668***	0.662***
Neck thickness	-0.085	0.203	0.238*	0.253*	1.000	0.325**	0.031	0.077
Average bulb weight	0.517***	0.652***	0.874***	0.714***	0.325***	1.000	0.792***	0.823***
Yield per plot	0.591***	0.595***	0.824***	0.668***	0.031	0.792***	1.000	0.924***
Yield per hectare	0.625***	0.600***	0.822***	0.662***	0.077	0.823***	0.924***	1.000

*, **, *** significant at *P* ≤ 0.05, 0.01, 0.001 respectively.

### Nutritional parameters

Thirty-one genotypes were evaluated for major biochemical and mineral traits. Significant genotypic variability was observed for all the studied traits indicating substantial scope for trait-specific selection and quality improvement in onion breeding programs. The total phenol content was ranged from 172.76–446.73 mg/100 g FW, with DOGR-2101 recording the highest phenolic content, whereas the lowest value for total phenols was observed in RHRO-2022-2 (**Table 4**). Pyruvic acid, an important indicator of onion pungency, ranged from 1.33–11.65 µmol/g FW, with the highest pungency recorded in DOGR-2110 and the lowest in DOGR-2108.

**Table 4:** Phytochemical estimation and nutritional analysis of 31 red onion genotypes.

Sr. no.	Name of genotypes	Total phenols (mg/100 g FW)	Pyruvic acid (µmol/g FW)	Flavonoids (mg/Kg FW)	FRAP (µmol /100 g FW)	Anthocyanin (mg/100 g DW)	Total sugars (%)	Reducing sugars (%)	Non-reducing sugars (%)	TSS (°brix)	Moisture content (%)	Dry Matter (%)
1	DOGR-2101	446.73^a^	8.25^de^	359.19^fg^	4200.72^b^	21.95^c^	5.47^d^	2.01^hijkl^	3.46^cde^	12.3^bcde^	86.67^ab^	13.33^ab^
2	DOGR-2102	315.07^fghi^	5.55^ijk^	318.83^gh^	1378.11^no^	2.87^m^	4.72^fgh^	2.60^cdef^	2.12^hijk^	12.1^bcde^	86.28^ab^	13.72^ab^
3	DOGR-2103	285.51^hijklm^	8.83^cd^	248.86^i^	2223.98i^jkl^	44.18^a^	4.79^fgh^	2.00^hijkl^	0.42^s^	10.8^def^	85.36^b^	14.64^a^
4	DOGR-2104	304.61^ghijk^	8.49^cd^	153.31^j^	1975.85^lm^	33.06^b^	3.53^k^	2.64^cdef^	0.89^qrs^	11.3^bcdef^	86.25^ab^	13.75^ab^
5	DOGR-2105	362.23^cde^	4.07^l^	453.51^b^	3892.35^b^	11.27^fghi^	6.76^a^	3.64^a^	3.12^cdef^	14.3^a^	87.75^ab^	12.25^ab^
6	DOGR-2106	318.31^fgh^	4.58^jkl^	627.07^a^	3260.06^cde^	9.08^hijk^	3.66^jk^	1.68l^m^	1.98^ijkl^	8.3^g^	88.31^ab^	11.69^ab^
7	DOGR-2107	362.21^cde^	2.69^m^	248.00^i^	2662.75^fghi^	4.97^klm^	2.86^lm^	1.44^mn^	1.41^lmnopq^	12.1^bcde^	87.86^ab^	12.14^ab^
8	DOGR-2108	364.72^cde^	1.33^n^	435.09^b^	2813.05^efgh^	12.75^defgh^	4.56^ghi^	2.66^cde^	1.90^jklmn^	12.3^bcd^	86.91^ab^	13.09^ab^
9	DOGR-2109	360.88^cde^	3.84^l^	344.91^fg^	3277.11^cd^	9.66^ghij^	3.69^jk^	1.74^klm^	1.95^ijklm^	12.1^bcde^	88.14^ab^	11.86^ab^
10	DOGR-2110	425.71^ab^	11.65^a^	219.39^i^	2027.60^jklm^	3.51^m^	4.84^efg^	1.28^n^	3.56^cd^	11.3^bcdef^	87.31^ab^	12.69^ab^
11	DOGR-2111	348.76^def^	6.92^fg^	377.64^cdef^	2776.31^fgh^	14.31^def^	4.25^hi^	3.10^b^	1.16^pqr^	10.6^ef^	86.95^ab^	13.05^ab^
12	DOGR-2112	364.67^cde^	6.56^fghi^	360.90^efg^	3422.68^c^	8.80^hijk^	2.69^m^	1.69^klm^	0.99^qrs^	11.8^bcdef^	88.22^ab^	11.78^ab^
13	DOGR-2113	324.30^fg^	4.28^l^	253.26^i^	3088.85^cdef^	6.03^jklm^	5.44^d^	2.50^defg^	2.95^efg^	12.7^abc^	85.58^ab^	14.42^ab^
14	DOGR-2114	332.40^efg^	5.62^hij^	427.50^bc^	2893.47^defg^	4.21^lm^	4.08^ij^	2.32^efgh^	1.76^jklmno^	12.3^bcd^	85.45^b^	14.55^a^
15	DOGR-2115	374.31^cd^	10.29^b^	274.07^hi^	1714.26^mno^	23.60^c^	5.75^cd^	2.14^ghi^	3.61^c^	12.9^ab^	87.22^ab^	12.78^ab^
16	DOGR-2116	279.11^ijklm^	8.33^de^	363.67^defg^	4322.13^b^	15.88^de^	4.52^ghi^	2.59^cdef^	1.93^jklmn^	11.3^bcdef^	87.34^ab^	12.66^ab^
17	DOGR-2117	241.30^no^	7.31^efg^	161.15^j^	2088.04j^klm^	8.15^ijkl^	6.59^ab^	1.73^klm^	4.86^a^	12.5^bcd^	85.66^ab^	14.34^ab^
18	DOGR-2118	368.50^cd^	6.29^ghi^	252.68^i^	1672.77^mno^	3.76^m^	4.48^ghi^	2.29^fgh^	2.19^hij^	11.7^bcdef^	86.12^ab^	13.88^ab^
19	DOGR-2119	274.21^klmn^	8.74^cd^	342.46^fg^	3351.48^c^	11.86^efghi^	4.33^ghi^	2.72^cd^	1.61^klmnop^	10.9^def^	85.80^ab^	14.20^ab^
20	RHRO-2022-1	391.04^bc^	1.71^mn^	340.04^fg^	1763.94^mn^	11.82^efghi^	6.19^bc^	1.91^ijkl^	4.28^b^	10.9^def^	88.64^ab^	11.36^ab^
21	RHRO-2022-2	172.76^p^	4.53^kl^	242.61^i^	2434.08^hijk^	21.86^c^	3.32^kl^	2.11^hij^	1.21^opqr^	10.1^f^	86.96^ab^	13.04^ab^
22	RHRO-2022-3	250.17^mno^	4.59^jkl^	433.36^bc^	5538.31^a^	15.38^def^	2.42^m^	2.87^bc^	1.92^jklmn^	12.4^bcd^	86.30^ab^	13.70^ab^
23	RHRO-2022-4	232.71°	2.53^m^	235.30^i^	2466.51^ghij^	16.09^de^	4.71^gh^	2.05^hijk^	2.66^fgh^	12.3^bcde^	87.12^ab^	12.88^ab^
24	RHRO-2022-5	311.92^ghij^	9.53^bc^	237.00^i^	2394.17^hijkl^	15.01^def^	3.32^kl^	2.62^cdef^	0.69^rs^	11.9^bcde^	87.60^ab^	12.40^ab^
25	RHRO-2022-6	287.68^hijkl^	9.43^bc^	138.40^j^	1284.15°	16.57^d^	4.79^fgh^	2.22^ghi^	2.57^fgh^	10.8^def^	89.13^a^	10.87^b^
26	RHRO-2022-7	255.94^lmno^	4.28^l^	416.83^bcde^	1674.46^mno^	8.42^hijkl^	5.35^de^	3.32^efgh^	3.03^defg^	11.9^bcde^	86.59^ab^	13.41^ab^
27	RHRO-2022-8	416.96^ab^	8.51^cd^	247.59^i^	2727.50^fgh^	13.46^defg^	5.26^def^	3.91^a^	1.36^nopq^	11.3^bcdef^	87.11^ab^	12.89^ab^
28	RHRO-2022-9	253.87^lmno^	6.65^fgh^	573.45^a^	5246.67^a^	24.36^c^	6.16^bc^	1.78^jklm^	4.38^ab^	11.1^cdef^	87.21^ab^	12.79^ab^
29	RHRO-2022-10	277.80^jklm^	7.36^ef^	243.66^i^	1988.17^klm^	5.80^jklm^	3.71^jk^	2.33^efgh^	1.37^mnopq^	10.9^def^	88.09^ab^	11.91^ab^
30	Bhima Super	331.29^efg^	6.42^fghi^	355.43^fg^	4138.01^b^	14.86^def^	4.14^ij^	3.57^a^	0.56^s^	12.8^ab^	86.76^ab^	13.24^ab^
31	Bhima Dark Red	393.89^bc^	6.50^fghi^	418.66^bcd^	3335.34^cd^	29.35^b^	4.38^ghi^	1.87^ijkl^	2.51^ghi^	12.8^abc^	86.20^ab^	13.80^ab^
	Mean	323.54	6.31	325.93	2839.77	14.29	4.54	2.33	2.21	11.70	87.00	13.00
	SE	6.44	0.19	10.22	81.07	0.78	0.10	0.07	0.10	0.30	0.66	0.66

The superscripted lowercase letter denotes grouping of genotypes and the genotypes with same letters are not significantly different.

Flavonoid content was ranged from 138.40–627.07 mg/kg FW, with DOGR-2106 exhibiting the highest flavonoid accumulation while least was observed in RHRO-2022-6. Similarly, antioxidant capacity determined through FRAP assay ranged from 1284.15–5538.31 µmol/100 g, with the highest antioxidant capacity was observed in RHRO-2022–3 while, the least was observed in RHRO-2022-6. The anthocyanin content varied significantly among the evaluated onion genotypes ranging from 2.87–44.18 mg/100 g DW, while highest anthocyanin was recorded in DOGR-2103 while lowest in DOGR-2102. Higher anthocyanin and flavonoid accumulation observed in certain red onion genotypes may contribute to enhanced antioxidant potential and functional food value.

Significant variability was also observed for total sugars, reducing sugars, non-reducing sugars and TSS among the studied genotypes (**Table 4**). The higher concentration of total sugars was recorded in DOGR-2105, whereas lowest in DOGR-2103. Reducing sugars ranged from 1.28–3.91%, with RHRO-2022–8 recording the highest concentration and DOGR-2110 the lowest. Similarly, non-reducing sugars were varied from 0.42–4.86% with higher concentration in DOGR-2117 while least was noted in genotype DOGR-2103. The TSS varied from 8.3–14.3°Brix, and the maximum value was observed in DOGR-2105 and the least in DOGR-2106. Genotypes possessing higher sugar content and TSS may be advantageous for dehydration, processing, and flavor enhancement due to greater dry matter accumulation and improved bulb quality.

Moisture content, a key determinant of bulb freshness and storage, varied from 85.36–89.13%. The maximum value was observed in genotype RHRO-2022–6 and the least in DOGR-2103. In contrast, dry matter content ranged from 10.87–14.64%, with the highest accumulation recorded in DOGR-2103 and the lowest in RHRO-2022-6. The inverse relationship observed between moisture and dry matter content suggests that low-moisture; high dry-matter genotypes may possess superior storability and processing suitability under rainy season conditions.

### Mineral content

Significant genotypic variation was observed for macro- and micronutrient composition among the evaluated onion genotypes (**Table 5**). Nitrogen content ranged from 0.51–1.63%, with RHRO-2022–6 recording the highest nitrogen concentration, whereas the lowest value was observed in DOGR-2115. Phosphorus content varied from 0.30–0.54%, with DOGR-2119 recording the highest phosphorus concentration, followed by DOGR-2101 and DOGR-2114, whereas the lowest values were observed in DOGR-2105 and DOGR-2107. Potassium content ranged from 0.88–1.63%, with the highest accumulation recorded in DOGR-2111 and the lowest in DOGR-2113. Similarly, sulfur content varied from 0.25–0.63%, with DOGR-2114 and DOGR-2111 recording the highest sulfur concentration, whereas DOGR-2115 exhibited the lowest value. Considerable variability was also observed for other mineral constituents, including calcium, magnesium, copper, manganese, and boron. Calcium content was ranged from 0.39–0.81%, with Bhima Super, DOGR-2105 and Bhima Dark Red recording comparatively higher calcium accumulation. In contrast, magnesium content did not exhibit significant variation among the studied genotypes. Copper, manganese, and boron contents ranged from 8.20–31.60 ppm, 7.60–18.47 ppm, and 17.53–57.32 ppm, respectively. The observed variability in mineral accumulation among genotypes indicates substantial genetic diversity for nutrient uptake and partitioning, which may be exploited for mineral biofortification and nutritional quality improvement in onion breeding programs.

**Table 5:** Micronutrient analysis of 31 red onion genotypes.

Sr. No.	Name of genotypes	N (%)	P (%)	K (%)	S (%)	Ca (%)	Mg (%)	Cu (ppm)	Mn (ppm)	B (ppm)
1	DOGR-2101	1.25^def^	0.51^ab^	1.03^mn^	0.38^cdef^	0.69^abcde^	0.11	22.7^ef^	10.0^jklmn^	44.43^bc^
2	DOGR-2102	1.07^hij^	0.39^cdefgh^	1.19^h^	0.40^bcd^	0.64^defg^	0.10	22.3^f^	9.5^klmno^	33.40^ef^
3	DOGR-2103	1.33^de^	0.35^defghi^	1.08^kl^	0.29^efghi^	0.73^abcd^	0.09	20.6^g^	8.6^mno^	20.22^jk^
4	DOGR-2104	1.05^ij^	0.43^cde^	1.38^cd^	0.38^cdef^	0.68^bcdef^	0.11	24.5^d^	9.3^lmno^	26.88^hi^
5	DOGR-2105	1.02^jk^	0.30^i^	1.08^l^	0.40^bcd^	0.81^a^	0.12	17.7^hi^	15.4^bc^	28.15^gh^
6	DOGR-2106	1.33^de^	0.32^ghi^	1.12^jk^	0.36^cdefg^	0.51^hijk^	0.12	13.7^jk^	10.9^hijkl^	32.65^fg^
7	DOGR-2107	0.89^lm^	0.30^i^	1.09^kl^	0.50^b^	0.72^abcd^	0.10	12.5^klm^	13.1efg	42.13^cd^
8	DOGR-2108	1.33^de^	0.34^efghi^	1.20^h^	0.40^bcd^	0.51^hijk^	0.09	22.5^ef^	7.6°	33.23^ef^
9	DOGR-2109	1.00^jkl^	0.40^cdefg^	1.31^ef^	0.45^bc^	0.76^abcd^	0.10	12.7^kl^	9.9^jklmn^	49.45^b^
10	DOGR-2110	1.02^jk^	0.40^cdefg^	1.00^n^	0.38^cde^	0.64^defg^	0.09	22.2^f^	11.1^ghijkl^	23.48^hij^
11	DOGR-2111	1.36^cd^	0.42^cdef^	1.63^a^	0.63^a^	0.77^abc^	0.09	14.5^j^	11.5^fghijk^	44.90^bc^
12	DOGR-2112	1.17^fghi^	0.42^cdef^	1.47^b^	0.36^cdefg^	0.56^fghi^	0.13	27.4^bc^	8.3^no^	23.20^hij^
13	DOGR-2113	0.92^klm^	0.34^efghi^	0.88°	0.25^hi^	0.39^k^	0.11	8.5^n^	9.8^jklmn^	44.67^bc^
14	DOGR-2114	1.47^bc^	0.46^abc^	1.35^de^	0.63^a^	0.79^ab^	0.11	8.2^n^	10.4^ijklm^	44.83^bc^
15	DOGR-2115	0.51°	0.42^cde^	1.29^fg^	0.25^i^	0.40^k^	0.10	26.2^c^	9.5^klmno^	32.53^fg^
16	DOGR-2116	0.88^lm^	0.38^cdefghi^	1.02^n^	0.43^bc^	0.65^cdefg^	0.09	31.6^a^	7.7°	32.33^fg^
17	DOGR-2117	0.75^n^	0.40^cdefg^	1.17^hi^	0.39^cde^	0.71^abcd^	0.11	8.3^n^	10.3^ijklmn^	45.32^bc^
18	DOGR-2118	1.06^ij^	0.38^cdefghi^	1.19^h^	0.31^defghi^	0.44^ijk^	0.10	26.4^bc^	9.3^lmno^	24.08^hij^
19	DOGR-2119	1.21^efg^	0.54^a^	1.31^ef^	0.35^cdefghi^	0.44^ijk^	0.11	27.7^b^	9.1^lmno^	32.58^fg^
20	RHRO-2022-1	1.10^ghij^	0.33^fghi^	1.41^c^	0.26^ghi^	0.69^abcde^	0.11	23.7^de^	13.7^cde^	57.32^a^
21	RHRO-2022-2	1.58^ab^	0.39^cdefgh^	1.31^ef^	0.26^ghi^	0.43^jk^	0.10	22.7^ef^	12.7^efgh^	47.67^b^
22	RHRO-2022-3	1.11^ghij^	0.35^defghi^	1.26^g^	0.36^cdefgh^	0.49^hijk^	0.10	30.3^a^	13.2^def^	34.40^ef^
23	RHRO-2022-4	1.19^fgh^	0.36^defghi^	1.30^fg^	0.25^hi^	0.57^efgh^	0.10	12.2^lm^	16.7^ab^	37.40^def^
24	RHRO-2022-5	1.50^ab^	0.30^hi^	1.17^hi^	0.25^hi^	0.41^k^	0.11	18.3^h^	16.5^ab^	35.52^ef^
25	RHRO-2022-6	1.63^a^	0.36^defghi^	1.17^hi^	0.28^efghi^	0.47^hijk^	0.09	16.9^i^	18.5a	27.78^gh^
26	RHRO-2022-7	1.10^ghij^	0.32^ghi^	1.33^ef^	0.45^bc^	0.47^hijk^	0.11	8.5^n^	10.2^ijklmn^	23.83^hij^
27	RHRO-2022-8	1.16^fghi^	0.37^defghi^	1.20^h^	0.26^ghi^	0.44^ijk^	0.07	23.1^ef^	10.6^hijklm^	17.53^k^
28	RHRO-2022-9	1.10^ghij^	0.35^defghi^	1.31^f^	0.27^fghi^	0.55^ghij^	0.08	20.1^g^	12.2^efghi^	21.67^jk^
29	RHRO-2022-10	1.21^efg^	0.35^defghi^	1.14^ij^	0.31^defghi^	0.69^abcde^	0.10	12.7^kl^	15.3^bcd^	22.40^ijk^
30	Bhima Super	0.85^mn^	0.42^cde^	1.08^kl^	0.28^efghi^	0.81^a^	0.13	11.3^lm^	9.3^lmno^	24.02^hij^
31	Bhima Dark Red	0.82^mn^	0.35^defghi^	1.07^lm^	0.25^hi^	0.80^ab^	0.11	11.3^m^	11.7^efghij^	37.75^de^
	Mean	1.13	0.38	1.21	0.36	0.60	NS	18.6	11.35	33.41
	Standard error	0.02	0.02	0.01	0.02	0.02	0.01	0.25	0.37	0.91

The superscripted lowercase letter denotes grouping of genotypes and the genotypes with same letters are not significantly different.

### Identification of promising donors for bulb quality breeding

**Figure 2:**
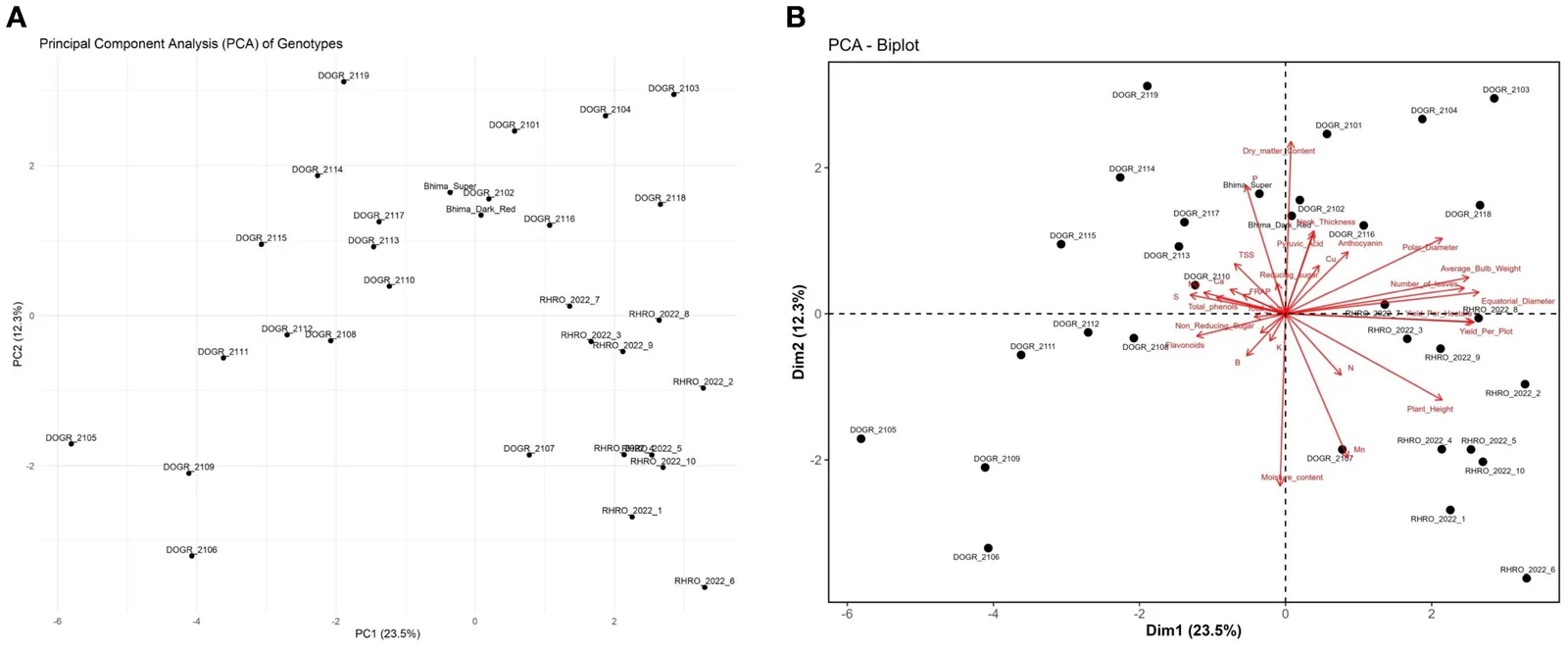
**(A)** Principal component analysis showing distribution of onion genotypes. **(B)** PCA biplot illustrating the relationship between genotypes and morphological, biochemical and mineral traits based on PC1 and PC2 in onion.

**Figure 3:**
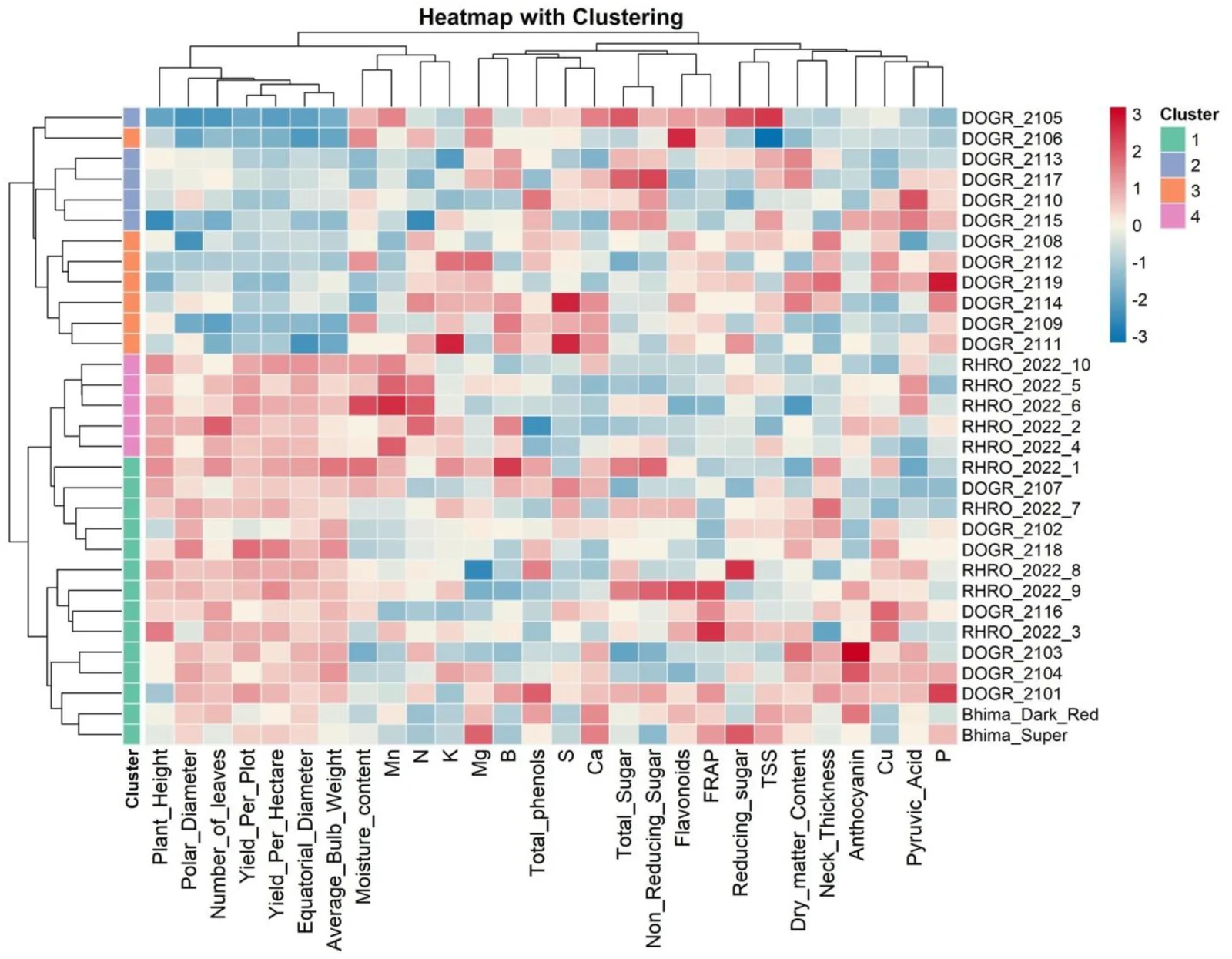
Heat map revealing patterns between the genotypic clusters and morphological, biochemical and mineral traits in thirty one red onion genotypes. (The clustering on the y axis represents the genotypic clusters and clustering on the top represents the traits).

The present study aimed to identify promising donors for bulb quality improvement and biofortification-oriented breeding programs through integrated evaluation of biochemical, nutritional, and mineral traits. Based on this, we categorized all the genotypes through Euclidean based clustering and PCA plot to identify the most promising donors. The PCA in the first two dimensions explained 35.8% of the total variation observed, with the first (PC1) and the second (PC2) components accounting for 23.5% and 12.3% of the total variation, respectively. Out of the total 28 traits studied, PC1 showed positive correlations with 14 traits while PC2 exhibits positive correlations with 13 traits. PCA clearly separated major trait groups, representing yield and quality related variation. It separates yield traits in PC1 and biochemical mineral traits in PC2 (**Figure 2A**). The PCA biplot further illustrated strong positive correlation among yield related traits and clustering of biochemical and mineral traits in PC2 (**Figure 2B**). Higher variability among the genotypes was observed for biochemical traits such as total phenols, flavonoids, phosphorous, potassium and yield related traits as evidenced by their strong contribution in PCA and distinct clustering patterns in heatmap (**Figure 3**).

In contrast, the relatively lower variability was observed for traits such as calcium and magnesium. These results led to identification of potential multi-trait donors i.e., DOGR-2103 for yield, total phenols, flavonoids, FRAP and mineral traits (N, P and K), DOGR-2104 and DOGR-2101 for combined yield and quality associated traits, DOGR-2118 and Bhima Dark Red for yield and antioxidant traits. For quality and mineral improvement, DOGR-2119, DOGR-2114, DOGR-2113 and DOGR-2115 were identified as donors for biochemical and mineral traits including phenols, flavonoids and macro nutrients. For improving yield, RHRO-2022-6, RHRO-2022–2 and RHRO-2022–10 were identified as single trait donors. The genotypes DOGR-2105 and DOGR-2110 were identified for processing and pungency while DOGR-2108 was identified as a potential donor for mineral biofortification, which may deploy in further breeding programs.

### Molecular characterization

SSR marker-based molecular characterization was performed to assess genetic diversity among the evaluated genotypes. A total of 50 SSR markers were screened, of which 35 markers showed amplification, with clear and reproducible banding patterns (**Supplementary Figures 3A, B**. Among the amplified markers, five highly polymorphic markers namely ACM-05 (0.62), ACM-06 (0.68), ACM-68 (0.80), ACM-45 (0.54), and ACM-09 (0.57), exhibited high polymorphism with polymorphic information content (PIC) values greater than 0.50, indicating their superior discriminatory power for diversity analysis (**Table 1**). The PIC values ranged from 0.06 to 0.80, indicating moderate genetic diversity among the studied onion genotypes, which may be attributed to the predominance of cultivated breeding lines and released varieties included in the present investigation.

**Figure 4:**
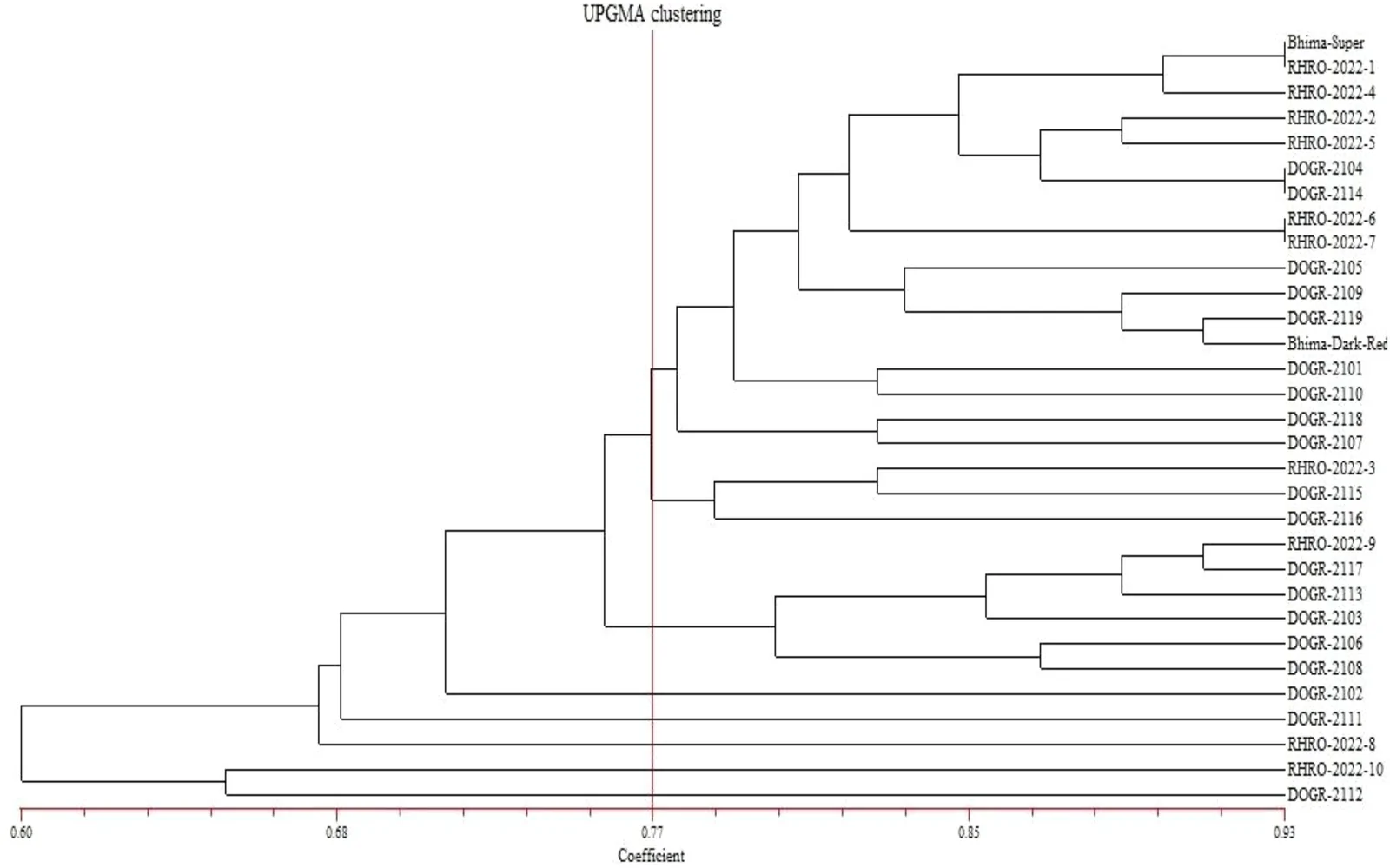
SSR based dendrogram of 31 genotypes.

To investigate relationships among the evaluated genotypes, we constructed an unweighted pair-group method with arithmetic mean dendrogram based on SSR marker data (**Figure 4**). The genetic similarity coefficient among genotypes ranged from 0.60 to 0.93, with an average value of 0.77. The cluster analysis performed at a similarity coefficient threshold of 0.77 grouped the thirty-one onion genotypes into eight major clusters, with clusters I, II, and III further subdivided into two sub-clusters each, indicating the presence of considerable genetic divergence among the evaluated genotypes.

### Cluster analysis

Cluster analysis based on SSR marker data grouped the evaluated onion genotypes into distinct clusters, reflecting substantial genetic diversity among the studied germplasm (**Figure 4**). At a similarity coefficient threshold of 0.77, the UPGMA dendrogram classified the thirty-one genotypes into eight major clusters. Cluster I was further subdivided into two sub-clusters, with sub-cluster Ia comprising fifteen genotypes and sub-cluster Ib containing two genotypes. Similarly, Cluster II consisted of two sub-clusters, namely IIa and IIb, comprising two and one genotype, respectively. Cluster III also exhibited two sub-clusters, with four genotypes grouped in IIIa and two genotypes in IIIb, whereas clusters IV, V, VI, VII, and VIII each contained a single genotype. The distribution of genotypes into distinct clusters and sub-clusters indicated considerable genetic divergence among the evaluated onion lines. Detailed genotype composition within each cluster is presented in **Table 6**. The observed clustering pattern further suggested the existence of genetically distinct groups that may serve as potential parental resources for hybridization and trait-specific breeding programs.

**Table 6:** Cluster analysis of red onion genotypes.

Cluster	Sub-cluster	Number of genotypes	Genotypes
I	Ia	15	Bhima Super, RHRO-2022-1, RHRO-2022-4, RHRO-2022-2, RHRO-2022-5, DOGR-2104, DOGR-2114, RHRO-2022-6, RHRO-2022-7, DOGR-2105, DOGR-2109, DOGR-2119, Bhima Dark Red, DOGR-2101, DOGR-2110
	Ib	2	DOGR-2118, DOGR-2107
II	IIa	2	RHRO-2022-3, DOGR-2115
	IIb	1	DOGR-2116
III	IIIa	4	RHRO-2022-9, DOGR-2117, DOGR-2113, DOGR-2103
	IIIb	2	DOGR-2106, DOGR-2108
IV		1	DOGR-2102
V		1	DOGR-2111
VI		1	RHRO-2022-8
VII		1	RHRO-2022-10
VIII		1	DOGR-2112

### Jaccard’s similarity coefficient

Jaccard’s similarity coefficient analysis based on SSR-amplified polymorphic fragments provided detailed insights into the genetic relationships among the evaluated onion genotypes. Pairwise similarity coefficients ranged from 0.43–0.93 (**Supplementary Table 2**), indicating the presence of substantial genetic diversity among the studied genotypes. The highest similarity coefficient (0.935) was observed between Bhima Super and RHRO-2022-1, suggesting close genetic relatedness, whereas the lowest similarity coefficient was recorded between RHRO-2022–10 and DOGR-2117, indicating maximum genetic divergence among the evaluated genotypes. Most genotype combinations exhibited moderate to high similarity coefficients (0.65–0.85), reflecting the presence of related sub-groups within the germplasm while still maintaining appreciable population-level diversity. The wide range of similarity coefficients observed in the present study highlights the potential utility of genetically divergent genotypes for broadening the genetic base in onion breeding programs.

## Discussion

The substantial variability observed among the evaluated onion genotypes for bulb and yield-related traits indicates the presence of considerable genetic diversity that can be effectively exploited in rainy season exclusive onion crop improvement programs. High GCV and PCV estimates recorded for average bulb weight, neck thickness, yield per plot and yield per hectare suggest the existence of wider genetic variability and greater opportunity for effective phenotypic selection of these economically important traits. Similar findings have been reported by Dwivedi et al. (2017) and Khosa and Dhatt (2013), who also documented comparatively higher variability for bulb yield and neck-related traits in onion germplasm. The high GCV values for the mentioned traits show ample scope of improvement through selection method. In contrast, plant height and TSS exhibited relatively lower GCV and PCV whereas moderate variability was observed for both polar diameter and number of leaves per plant. Low variability for vegetative traits may indicate comparatively narrow genetic divergence and stronger environmental influence on trait expression, which could reduce the efficiency of direct selection. These results favor the selection for plants/genotypes with moderate canopy with higher bulb yield. Furthermore, the relatively narrow differences between GCV and PCV observed for most traits depict limited environmental influence and predominant genetic control over phenotypic expression. High heritability coupled with high genetic advance observed for most bulb and yield-related traits indicates the predominance of additive gene action, thereby suggesting greater effectiveness of selection for these traits in breeding programs. Conversely, traits exhibiting high heritability associated with low genetic advance, such as TSS and plant height, may be governed by non-additive gene effects or epistatic interactions, thereby limiting the expected genetic gain through direct phenotypic selection. Similar observations have been reported in earlier onion variability studies, emphasizing the importance of combining heritability estimates with genetic advance for effective selection decisions.

Remarkable genetic variation was reported for phenols, flavonoids, anthocyanins, antioxidant activity, and pungency among the evaluated genotypes, highlighting the strong influence of genotype and seasonal environment on biochemical quality traits in onion. The phenolic content recorded in the present investigation was comparable with earlier reports in rainy season germplasm (Islam et al., 2017). Previous studies by Lee et al. (2015) and Brahimi et al. (2022) also reported comparatively lower phenolic accumulation in white onions than in red and yellow onion types, supporting the higher antioxidant potential generally associated with pigmented red color bulbs. Enhanced accumulation of phenolic compounds and flavonoids under rainy season conditions may reflect stress-induced activation of secondary metabolite biosynthesis pathways, particularly under fluctuating temperature and intermittent episodes of high moisture availability followed by less moisture availability prevalent during the South-West monsoon season in Indian sub-continent (Islam et al., 2017; Dubey et al., 2020). Such secondary metabolites play important roles in reactive oxygen species scavenging and oxidative stress mitigation, thereby contributing to improved nutraceutical quality and functional food value of onion bulbs (Rudrapal et al., 2022).

The remarkable variation observed for pyruvic acid content further indicated the existence of distinct pungency classes among the evaluated genotypes. Similar pungency classifications based on pyruvic acid accumulation have been reported by Dhumal et al. (2007); Islam et al. (2019), and Gupta et al. (2025a). Variation in pungency is primarily associated with differential sulfur metabolism and its accumulation within the plant, which directly influence consumer preference, culinary utility, and dehydration suitability in onion (Randle, 1992; Randle et al., 1995). Genotypes possessing moderate to high pungency may therefore be useful for processing and storage-oriented breeding programs. Moderate to high pungency acts as a proxy for superior post-harvest longevity and industrial processing efficiency due to high total soluble solids and low moisture content, as the current study confirms. These traits, together with the sulfur containing ACSOs and their hydrolysis by the enzyme allinase to produce thiosulfinates and volatile sulfur compounds, contribute to improved storability and reduced post-harvest deterioration (Bhandari et al., 2026). In similar manner, anthocyanin and flavonoid content showcased marked variability corroborates earlier reports of substantial diversity in antioxidant-associated pigments and flavonoid compounds (Dalamu et al., 2010; Gorrepati et al., 2024). The accumulation of cyanidin-based anthocyanins and flavonoids in the outer bulb scales of pigmented genotypes (Slimestad et al., 2007; Rhodes and Price, 1996), contributes significantly to bulb pigmentation and antioxidant activity. The positive association between anthocyanin accumulation and antioxidant potential observed in the present study further emphasizes the importance of red onion germplasm as a valuable genetic resource for developing nutritionally enriched cultivars with enhanced health-promoting properties.

In addition, the present study pronounced the considerable variability for sugars, TSS, moisture, and dry matter content indicating substantial diversity for bulb quality and processing-related traits. The values recorded for total sugars, reducing sugars, non-reducing sugars, moisture content, and TSS were largely consistent with earlier reports in onion germplasm in India (Behera et al., 2017; Manjunathagowda et al., 2019; Gupta et al., 2025a), thereby supporting the reliability of the observed variation. The positive association among sugars, TSS, and dry matter content observed in the present study suggests enhanced assimilate accumulation and carbohydrate partitioning toward bulb tissues. Higher TSS and sugar accumulation are particularly desirable for dehydration and processing industries because they improve bulb recovery, flavor intensity, and processing efficiency. Similar relationships between TSS and carbohydrate accumulation have also been reported by Gogoi et al. (2023).

Diverging from these results, moisture content exhibited a strong negative association with dry matter and sweetness-related traits, indicating a physiological trade-off between water accumulation and assimilate concentration in bulbs. Similar variability for moisture and dry matter content in onion has also been reported by Priyadarshini et al. (2020). One of the major breeding challenges in rainy season onion is the development of high-yielding genotypes possessing low moisture and high dry matter content, as such genotypes are generally associated with prolonged storability, reduced post-harvest deterioration, improved shelf life, and superior processing suitability. The wide variation observed for these compositional traits under rainy season conditions highlights the importance of season-specific evaluation for identifying onion genotypes suitable for dehydration, storage, and value-added processing applications.

The significant diversity in macro- and micronutrient profiles across the studied onion genotypes underscores excellent foundation for mineral biofortification via selective breeding. The -concentrations recorded in the present study were generally comparable with earlier reports in onion germplasm (Bello et al., 2013; Akinwande and Olatunde, 2015), thereby confirming the existence of considerable diversity for nutrient accumulation traits. Variation in nitrogen, phosphorus, potassium, sulfur, calcium, and micronutrient content among genotypes may be attributed to differences in nutrient uptake efficiency, translocation, assimilation, and storage within bulb tissues. Similar enhancement in bulb mineral composition under favorable nutrient regimes has been reported by Thangasamy et al. (2024), suggesting strong interaction between genetic potential and nutrient utilization efficiency. The observed genotypic variability in mineral profiles underscores the need to breed climate-resilient nutrient use efficient genotypes with optimized root architectures and superior nutrient translocation traits. The comparatively higher accumulation of certain micronutrients, particularly boron and manganese, observed in selected genotypes may indicate superior nutrient acquisition and partitioning ability under rainy season conditions. In contrast, earlier reports by Abubakar et al. (2025) documented comparatively lower copper concentrations in onion bulbs, highlighting the influence of genotype, environmental conditions, and edaphic factors on micronutrient accumulation patterns. The observed variability in mineral composition is particularly important from a nutritional point of view, as identification of nutrient-dense onion genotypes may facilitate the development of biofortified cultivars capable of addressing micronutrient deficiencies through regular dietary consumption. Multiple biofortified varieties across various crops including iron-rich pearl millet (Dhanashakti) in India (Rai et al., 2014), provitamin A orange-fleshed sweet potato in Africa (Low et al., 2017), zinc wheat across South Asia (Ram et al., 2024), and zinc rice in Bangladesh (Rao et al., 2020), have successfully been released and adopted by millions of farmers. The present study demonstrated the effectiveness of multivariate approaches in revealing complex relationships among yield, biochemical and mineral traits in onion genotypes.

The separation of yield related traits along PC1 and biochemical and mineral traits along PC2 indicates distinct but complementary trait groups, suggesting the possibility of simultaneous improvement through targeted breeding. The PCA biplot further confirmed strong positive associations among yield traits and clustering of biochemical and mineral traits reflecting coordinated expression of quality attributes. The heat map clustering supported these findings by grouping genotypes with similar trait profiles, thereby highlighting the genetic divergence among genotypes. Higher variability observed for biochemical traits and key minerals such as phosphorous and potassium suggests their suitability for selection, whereas relatively lower variability for calcium and magnesium indicates their stability across genotypes. The identification of genotypes such as DOGR-2103, DOGR-2104 and DOGR-2101 as multi-trait donors highlights their potential for improving yield, quality and nutritional traits. The presence of distinct donor groups for yield, quality and mineral traits further provides opportunities for designing efficient breeding strategies through hybridization. Overall, the integration of PCA, biplot and heatmap clustering analyses enabled precise identification of superior donor genotypes for developing high yielding and nutritionally enriched onion cultivars.

SSR marker analysis revealed moderate levels of genetic diversity among the evaluated onion genotypes, as reflected by the observed PIC values and clustering patterns. The average PIC value obtained in the present investigation was comparable with the findings of Lyngkhoi et al. (2021), who also reported moderate to high levels of polymorphism among onion genotypes using under Indian conditions using both short day and long day environments using SSR markers. The high discriminatory power exhibited by several ACM-series markers further confirms their effectiveness for genetic diversity analysis and parental selection in onion breeding programs. The clustering and similarity analyses clearly differentiated genetically diverse genotypes, thereby facilitating identification of potential parental combinations for hybridization and trait introgression. In particular, RHRO-2022–10 and DOGR-2107 exhibited substantial genetic divergence and may therefore serve as promising parental lines for broadening the genetic base of rainy season onion breeding programs. The integration of molecular diversity analysis with biochemical and nutritional profiling provides a more robust framework for selecting genetically diverse and trait-specific donor genotypes, thereby supporting marker-assisted selection and future trait mapping studies in onion (Benke et al., 2025).

The observed variation in antioxidant compounds, mineral accumulation, and quality-associated traits further suggests the possible involvement of complex genotype × environment interactions influencing trait expression under rainy season conditions. The identified donor genotypes and trait associations generated in the present study provide valuable baseline resources for future onion breeding programs targeting higher yield under rainy season conditions, enhanced nutritional quality for food security, improved storability which is still for rainy season varieties, and superior processing suitability under rainy season agro-climatic conditions. In addition, Future breeding efforts should focus on combining high yield potential with low moisture, high dry matter, enhanced antioxidant activity, and superior mineral composition to develop nutritionally enriched rainy season onion cultivars suitable for prolonged storage and processing applications.

## Conclusions

The present study demonstrated substantial morphological, biochemical, mineral, and molecular variability among rainy season onion genotypes, highlighting significant opportunities for developing nutritionally enriched and processing-suitable cultivars. The integrated evaluation of antioxidant compounds, sugars, dry matter, mineral composition, and molecular diversity enabled precise identification of superior donor genotypes possessing desirable combinations of quality-associated traits. The high heritability coupled with high GAM recorded for several bulb and yield-related traits indicates considerable scope for effective phenotypic selection in future breeding programs. The integration of molecular diversity analysis with phenotypic and nutritional profiling strengthens the reliability of parental selection and provides a robust framework for future marker-assisted breeding efforts in onion. Genetically distinct genotypes identified through SSR analysis may therefore serve as valuable breeding resources for broadening the genetic base and developing superior recombinants possessing enhanced bulb quality and climatic adaptability. Although the present investigation generated valuable baseline information for rainy season onion improvement, the study was conducted under a specific environmental condition, and multi-location evaluation would further strengthen understanding of genotype × environment interactions influencing nutritional and quality traits. Future research integrating genomics, metabolomics and trait-linked molecular markers may help elucidate the genetic mechanisms governing antioxidant accumulation, mineral partitioning, storability and processing quality in onion bulbs.

## Data Availability

The original contributions presented in the study are included in the article/**Supplementary Material**. Further inquiries can be directed to the corresponding author.
