# Supplementary material for: Deciphering trait diversity in rainy-season onion: a comprehensive evaluation of morphological, nutritional, biochemical, and molecular attributes
Source: Front Plant Sci. 2026 Aug 12;17:1900180. doi: 10.3389/fpls.2026.1900180 (PMC13506848; doi:10.3389/fpls.2026.1900180)
Supplement: Supplementary file 1 [file DataSheet1.docx]

|  | GCV (%) | PCV (%) | Heritability | Genetic advance (%) (i=5%) |
| --- | --- | --- | --- | --- |
| Plant height | 7.74 | 9.47 | 66.8 | 13.04 |
| Number of leaves per plant | 13.39 | 17.95 | 55.7 | 20.59 |
| Equatorial diameter | 23.97 | 25.73 | 86.8 | 46.01 |
| Polar diameter | 13.25 | 14.73 | 81 | 24.57 |
| Neck thickness | 26.07 | 30.31 | 74 | 46.2 |
| Average bulb weight | 37.72 | 38.24 | 97.3 | 76.64 |
| Yield per plot | 45.66 | 47.06 | 94.2 | 91.28 |
| Yield per hectare | 40.04 | 41.14 | 94.7 | 80.28 |
| TSS | 8.79 | 9.85 | 79.7 | 16.17 |

**Supplementary figure 1:** Histogram depicting the estimates of phenotypic, genotypic and error coefficient of variation, heritability and genetic advance for various bulb and plant traits of 31 onion genotypes

(PCV- Phenotypic Coefficient of Variance, GCV- Genotypic Coefficient of Variance, H^2^- heritability, GA- Genetic advance)

| **DOGR-2101** |  | **DOGR-2102** |  | **DOGR-2103** |  | **DOGR-2104** |
| --- | --- | --- | --- | --- | --- | --- |
|  |  |  |  |  |  |  |
| **DOGR-2105** |  | **DOGR-2106** |  | **DOGR-2107** |  | **DOGR-2108** |
|  |  |  |  |  |  |  |
| **DOGR-2109** |  | **DOGR-2110** |  | **DOGR-2111** |  | **DOGR-2112** |

**Supplementary figure 2(A):** Photographs of red onion genotypes

| **DOGR-2113** |  | **DOGR-2114** |  | **DOGR-2115** |  | **DOGR-2116** |
| --- | --- | --- | --- | --- | --- | --- |
|  |  |  |  |  |  |  |
| **DOGR-2117** |  | **DOGR-2118** |  | **DOGR-2119** |  | **RHRO-2022-1** |
|  |  |  |  |  |  |  |
| **RHRO-2022-2** |  | **RHRO-2022-3** |  | **RHRO-2022-4** |  | **RHRO-2022-5** |

**Supplementary Figure 2(B):** photographs of red onion genotypes

| **RHRO-2022-6** |  | **RHRO-2022-7** |  | **RHRO-2022-8** |  | **RHRO-2022-9** |
| --- | --- | --- | --- | --- | --- | --- |
|  |  |  |  |  |  |  |
| **RHRO-2022-10** |  | **Bhima Super** |  | **Bhima Dark Red** |  |  |

**Supplementary Figure 2(C):** photographs of red onion genotypes

| L - Ladder | 1. Bhima super | 2.RHRO-2022-1 | 3. RHRO-2022-2 | 4. RHRO-2022-3 |
| --- | --- | --- | --- | --- |
| 5.RHRO-2022-4 | 6. RHRO-2022-5 | 7.RHRO-2022-6 | 8.RHRO-2022-7 | 9. RHRO-2022-8 |
| 10. RHRO-2022-9 | 11.RHRO-2022-10 | 12.DOGR-2101 | 13.DOGR-2102 | 14.DOGR-2103 |
| 15.DOGR-2104 | 16. DOGR-2110 | 17. DOGR-2114 | 18.DOGR-2115 | 19. DOGR-2116 |
| 20. DOGR-2117 | 21. DOGR-2118 | 22.DOGR-2112 | 23.DOGR-2105 | 24. DOGR-2106 |
| 25. DOGR-2111 | 26.DOGR-2113 | 27.DOGR-2107 | 28. DOGR-2109 | 29. DOGR-2108 |
| 30.DOGR-2119 | 31. Bhima Dark Red | | | |

**Supplementary Figure 3(A):** Representative agarose gel electrophoretic patterns of SSR marker (ACM 53)

| L - Ladder | 1. Bhima super | 2.RHRO-2022-1 | 3. RHRO-2022-2 | 4. RHRO-2022-3 |
| --- | --- | --- | --- | --- |
| 5.RHRO-2022-4 | 6. RHRO-2022-5 | 7.RHRO-2022-6 | 8.RHRO-2022-7 | 9. RHRO-2022-8 |
| 10. RHRO-2022-9 | 11.RHRO-2022-10 | 12.DOGR-2101 | 13.DOGR-2102 | 14.DOGR-2103 |
| 15.DOGR-2104 | 16. DOGR-2110 | 17. DOGR-2114 | 18.DOGR-2115 | 19. DOGR-2116 |
| 20. DOGR-2117 | 21. DOGR-2118 | 22.DOGR-2112 | 23.DOGR-2105 | 24. DOGR-2106 |
| 25. DOGR-2111 | 26.DOGR-2113 | 27.DOGR-2107 | 28. DOGR-2109 | 29. DOGR-2108 |
| 30.DOGR-2119 | 31. Bhima Dark Red | | | |

**Supplementary Figure 3(B):** Representative agarose gel electrophoretic patterns of SSR marker (ACM 78)
