# Supplementary material for: Deciphering trait diversity in rainy-season onion: a comprehensive evaluation of morphological, nutritional, biochemical, and molecular attributes
Source: Front Plant Sci. 2026 Aug 12;17:1900180. doi: 10.3389/fpls.2026.1900180 (PMC13506848; doi:10.3389/fpls.2026.1900180)
Supplement: Supplementary file 2 [file Table1.docx]

**Supplementary Table 1: List of 31 red onion genotypes used in the study**

| **Sr. No.** | **Name of Genotype** | **Source** |
| --- | --- | --- |
| 1 | DOGR-2101 | ICAR-DOGR, Pune, Maharashtra |
| 2 | DOGR-2102 | ICAR-DOGR, Pune, Maharashtra |
| 3 | DOGR-2103 | ICAR-DOGR, Pune, Maharashtra |
| 4 | DOGR-2104 | ICAR-DOGR, Pune, Maharashtra |
| 5 | DOGR-2105 | ICAR-DOGR, Pune, Maharashtra |
| 6 | DOGR-2106 | ICAR-DOGR, Pune, Maharashtra |
| 7 | DOGR-2107 | ICAR-DOGR, Pune, Maharashtra |
| 8 | DOGR-2108 | ICAR-DOGR, Pune, Maharashtra |
| 9 | DOGR-2109 | ICAR-DOGR, Pune, Maharashtra |
| 10 | DOGR-2110 | ICAR-DOGR, Pune, Maharashtra |
| 11 | DOGR-2111 | ICAR-DOGR, Pune, Maharashtra |
| 12 | DOGR-2112 | ICAR-DOGR, Pune, Maharashtra |
| 13 | DOGR-2113 | ICAR-DOGR, Pune, Maharashtra |
| 14 | DOGR-2114 | ICAR-DOGR, Pune, Maharashtra |
| 15 | DOGR-2115 | ICAR-DOGR, Pune, Maharashtra |
| 16 | DOGR-2116 | ICAR-DOGR, Pune, Maharashtra |
| 17 | DOGR-2117 | ICAR-DOGR, Pune, Maharashtra |
| 18 | DOGR-2118 | ICAR-DOGR, Pune, Maharashtra |
| 19 | DOGR-2119 | ICAR-DOGR, Pune, Maharashtra |
| 20 | RHRO-2022-1 | MPKV, Rahuri, Maharashtra |
| 21 | RHRO-2022-2 | MPKV, Rahuri, Maharashtra |
| 22 | RHRO-2022-3 | MPKV, Rahuri, Maharashtra |
| 23 | RHRO-2022-4 | MPKV, Rahuri, Maharashtra |
| 24 | RHRO-2022-5 | MPKV, Rahuri, Maharashtra |
| 25 | RHRO-2022-6 | MPKV, Rahuri, Maharashtra |
| 26 | RHRO-2022-7 | MPKV, Rahuri, Maharashtra |
| 27 | RHRO-2022-8 | MPKV, Rahuri, Maharashtra |
| 28 | RHRO-2022-9 | MPKV, Rahuri, Maharashtra |
| 29 | RHRO-2022-10 | MPKV, Rahuri, Maharashtra |
| 30 | Bhima Super | Released variety |
| 31 | Bhima Dark Red | Released variety |
